# SPINE20 recommendations 2025: Sustainable spine care for all

**DOI:** 10.1016/j.bas.2025.105886

**Published:** 2025-12-05

**Authors:** Adriaan J. Vlok, Koji Tamai, Suhail S. Alassiri, Thomas R. Blattert, Marco A. Campello, Robert N. Dunn, Komal Kamra, Kazuya Kitamura, Lisa C. Roberts, Carlo Ruosi, Francois D.V. Theron, Carlos Tucci, Ratko Yurac, Bridget Bromfield, Mufudzi Chihambakwe, Quinette A. Louw, Danella Lubbe, Almero Oosthuizen, André Bussières, Harvinder S. Chhabra, Pierre Côté, Giuseppe Costanzo, Bambang Darwono, Scott Haldeman, Jeremie S. Larouche, Eric J. Muehlbauer, Johan G. Van Lerbeirghe, Hana I. Alsobayel, Joerg Franke, Paulo Pereira, Michael Piccirillo, Sanjay Wadhwa, Karsten Wiechert, André L.F. Andújar, Luis E. Carelli, Alexandre F. Cristante, Cristiano M. Menezes, Robert Meves, Luciano MR. Rodrigues, Marcelo I. Risso-Net, Sami AlEissa

**Affiliations:** a Department of Neurosurgery, University of Stellenbosch, Cape Town, South Africa; b Department of Orthopaedic Surgery, Osaka Metropolitan University, Osaka, Japan; c Department of Orthopaedic Surgery, King Saud bin Abdulaziz University for Health Sciences, Riyadh, Saudi Arabia; d Interdisciplinary Spine Center, Klinikum Ingolstadt, Ingolstadt, Germany; e Department of Orthopaedic Surgery, NYU School of Medicine, New York, USA; f Department of Orthopaedic Surgery, University of Cape Town and Groote Schuur Hospital, Cape Town, South Africa; g The Spinal Foundation, Delhi, India; h Department of Orthopaedic Surgery, National Defense Medical College, Saitama, Japan; i School of Health Sciences, University of Southampton, Southampton, UK; j Public Health Department, Federico II University Napoli, Napoli, Italy; k Department of Orthopaedic Surgery, University of Pretoria, Pretoria, South Africa; l CEPPS-Centro de Estudos e Promoção de Pol'ıticas em Sa'ude, Einstein Hospital Israelita, Sao Paulo, Brazil; m Orthopedic Department, Clinica Alemana, Universidad del desarrollo, Santiago, Chile; n Chiropractor, Chiropractic Association of South Africa, Centurion, South Africa; o Department of Chiropractic, Durban University of Technology, Durban, South Africa; p Physiotherapy Department, Stellenbosch University, Cape Town, South Africa; q Chiropractic, Chiropractic Association of South Africa, Cape Town, South Africa; r Clinical Service Improvement, Western Cape Department of Health and Wellness, Cape Town, South Africa; s Département chiropratique, Université du Québec à Trois-Rivières, Québec, Canada; t Spine department, Sri Balaji Action Medical Institute, New Delhi, India; u Institute for Disability and Rehabilitation Research, Faculty of Health Sciences, Ontario Tech University, Ontario, Canada; v Orthopedic department, Rome University Sapienza, Roma, Italy; w Department of Orthopedic Surgery, Gading Pluit Hospital, Jakarta, Indonesia; x Department of Neurology, University of California, Irvine, CA, USA; y Orthopaedic Department, University of Calgary, Calgary, Canada; z Executive Office, North American Spine Society, Chicago, USA; aa Orthopedic Department, AZ Sint Lucas Gent, Gent, Belgium; ab Department of Rehabilitation Sciences, King Saud University, Riyadh, Saudi Arabia; ac Department of Orthopedics, Klinikum Magdeburg gGmbH, Magdeburg, Germany; ad Neurosurgery Department, University Hospital São João, Porto, Portugal; ae Centre for Philanthropy Development, Blackwell Foundation, Zürich, Switzerland; af Department of Physical Medicine and Rehabilitation, All India Institute of Medical Sciences, New Delhi, India; ag Comprehensive Pain Program, Michel Spine Center, Hamburg, Germany; ah Pediatric Orthopedic Department, Hospital Infantil Joana de Gusmão, Florianópolis, Brazil; ai Spine Center, National Institute of Traumatology and Orthopaedics – INTO, Rio de Janeiro, Brazil; aj Department of Orthopedic Surgery, University of São Paulo, São Paulo, Brazil; ak Department of Locomotor Apparatus, Federal University of Minas Gerais, Belo Horizonte, Brazil; al Orthopedic Department, Santa Casa Spine Center, São Paulo, Brazil; am Einstein Hospital Israelita, São Paulo, Brazil; an Spine Surgery Division on Orthopedic Department, UNICAMP-Universidade Estadual de Campinas, Campinas, Brazil; ao Department of Orthopedics, King Abdulaziz Medical City, Riyadh, Saudi Arabia

**Keywords:** SPINE20, Sustainability, Public health, Occupational health & safety policy, Capacity building

## Abstract

Spine disorders remain a leading cause of disability worldwide, affecting over 900 million people and creating profound social and economic burden. In response, SPINE20, a global alliance of 38 professional societies, presents its 2025 policy recommendations under the theme “Sustainable Spine Care for All”.

Main recommendation; SPINE20 recommends G20 countries to implement sustainable evidence-based spine care models drawing on successful global programs considering particularly registries, incentivized health targets and public-private partnerships.

Focused on “Public health”; SPINE20 recommends G20 countries to integrate spine health into public health and primary care health policies by addressing the prevention and management of both communicable and non-communicable diseases, and strengthening public–private partnerships to achieve sustainable spine care.

Focused on “Occupational Health & Safety Policy”; SPINE20 recommends that G20 countries implement evidence-informed, work-focused interventions that address employee and workforce factors early, to reduce the social and economic impact of work loss and increase employability for people with spine disorders.

Focused on “Capacity Building”; SPINE20 recommends that G20 countries prioritize building capacity in spinal cord injury care by adopting evidence-based interventions such as the global initiatives supported by World Health Organization (WHO) in low- and middle-income countries and aligned with the WHO Rehabilitation 2030 Call to Action.

This paper serves as a summary of the recommendations. The complete set of SPINE20 2025 Recommendations, which is available in SPINE20 official web-site (https://spine20.net), was officially presented to Provincial Minister of Health and Wellness, Western Cape Government, during the SPINE20 Summit 2025. An official communication from the Western Cape Ministry of Health and Wellness subsequently confirmed formal acknowledgment of receipt of the recommendations.

## Highlights

• SPINE20 recommends G20 to adopt sustainable, evidence-based spine care models.
• Integrate spine health into public health, primary care, and prevention systems.
• Implement work-focused interventions to reduce disability and improve employability.
• Build national capacity for spinal cord injury care aligned with WHO initiatives.
• Promote registries, public-private partnerships, and outcome-based health targets.

## 1 Introduction

Spine disorders remain a leading cause of disability and reduced quality of life across the globe (Diseases and Injuries, 2020; Cieza et al., 2021; GBDLBP, 2023; Hoy et al., 2012). Current estimates indicate that the total number of individuals living with spine-related disorders approaches 900 million, and projections suggest this figure may exceed 1 billion by 2050 (GBDLBP, 2023; GBDNP, 2024; Lei et al., 2025; Lu et al., 2024). Despite remarkable advances in health sciences, musculoskeletal conditions—particularly low back and neck pain—continue to exert an immense burden on individuals, communities, and economies (Initiative USBaJ, 2021; Yong et al., 2022; World Health Organization). Affecting over 620 million individuals globally, low back and neck pain are not only the leading causes of disability, but also significant contributors to labor market exclusion, rising healthcare expenditure, and long-term social welfare dependency (Chou, 2021; Maher et al., 2017; Roux et al., 2005). Despite their significant socioeconomic toll, spine disorders continue to be overlooked in national health policies and global development agendas.

In response to these challenges, SPINE20 was launched in 2019 by four leading spine societies—EUROSPINE, the North American Spine Society, the German Spine Society, and the Saudi Spine Society. Since its inception, SPINE20 has grown into a global advocacy alliance, now representing 38 professional societies, across six continents (Table 1). SPINE20's mission is to provide evidence-informed policy recommendations to G20 nations, aiming to promote equitable, integrated, and sustainable spine care systems (AlEissa et al., 2021; Costanzo et al., 2022; Darwono et al., 2022; Chhabra et al., 2023; Menezes et al., 2025). By anchoring its proposals in both international frameworks such as the initiatives of World Health Organization (WHO), and the lived realities of the host country, SPINE20 delivers policy messages that are both globally relevant and locally actionable.

**Table 1:** Societies participating in SPINE20 (August 2025).

Category	Society Name	Country
Academic Entity	African Chiropractic Federation	South Africa
Patient Entity	Amar Seva Sangam	India
Academic Entity	Asociacion Mexicana de Cirujanos de Columna	Mexico
Academic Entity	Association of Spine Surgeons of India	India
Academic Entity	Australian Physiotherapy Association	Australia
Academic Entity	Brazilian Spine Society	Brazil
Academic Entity	Chandigarh Spinal Rehab	India
Academic Entity	Chilean Spine Society and Spine Committee of Chilean Orthopaedic and Traumatology Society SCHOT	Chile
Academic Entity	EUROSPINE	International
Academic Entity	German Spine Society	Germany
Academic Entity	Hellenic Spine Society	Greece
Academic Entity	Indian Association of Physical Medicine and Rehabilitation	India
Patient Entity	Indian Head Injury Foundation	India
Academic Entity	Indonesia Spine Society	Indonesia
Academic Entity	Italian Spine Society (SICV&GIS)	Italy
Patient Entity	Japanese Association for Patients with Spinal Ligament Ossification	Japan
Academic Entity	Japanese Society for Spine Surgery and Related Research	Japan
Patient Entity	Nina Foundation	India
Academic Entity	North America Spine Society	United States
Commercial Entity - Education Focused	NSPINE	Germany
Academic Entity	Renè Perdriolle Academy for Scoliosis Study	Italy
Academic Entity	Saudi Association of Neurological Surgery	Saudi Arabia
Academic Entity	Saudi Spine Society	Saudi Arabia
Academic Entity	Sociedad Iberolatinoamericana de Columna	Uruguay
Academic Entity	Society of Indian Physiotherapists	India
Academic Entity	Society of Spine Surgeons of Pakistan	Pakistan
Academic Entity	Spinal Cord Society	India
Academic Entity	Spine Society Delhi Chapter	India
Academic Entity	Spine Society of Belgium	Belgium
Patient Entity	The Ability People	India
Patient Entity	The Association of People with Disability	India
Patient Entity	The Chiropractic Association of South Africa	South Africa
Academic Entity	The Indonesian Association of Physical Medicine and Rehabilitation	Indonesia
Patient Entity	The Spinal Cord Injury Association	India
Patient Entity	The Spinal Foundation	India
Patient Entity	The South African Spine Society	South Africa
Academic Entity	World Federation of Chiropractic	Canada
Patient Entity	World Spine Care	United States

In 2025, SPINE20 convened in South Africa, a nation of rich cultural diversity and resilience but also one facing significant public health and social equity challenges (Louw et al., 2023). Spine-related disorders are a growing concern in South Africa, where disparities in access to rehabilitation, workforce reintegration, and preventive care mirror broader issues in healthcare delivery and social inclusion (Louw et al., 2023; Mazibuko et al., 2004). This year's G20 summit, also hosted in South Africa, adopted the theme “Solidarity, Equality, Sustainability”. In alignment with this, SPINE20 introduced the theme “**Sustainable Spine Care for All**” as its central concept of the summit and recommendations. This theme underscores the urgent need for scalable, context-sensitive strategies that prioritize prevention, timely and accurate diagnosis, early intervention, and continuity of care—particularly in under-resourced settings. It also highlights the importance of cross-sectoral collaboration involving governments, health systems, academia, and civil society to ensure that no individual is left behind due to spinal disability.

## 2 Methods to develop recommendations 2025

### 2.1 Authors

SPINE20 ‘Scientific Task Force’ members, from 26 international spine related societies, coordinated and developed the recommendations. The task forces involved multiprofessional panels including patient representatives, surgeons, rehabilitation clinicians, researchers, epidemiologists, primary care physicians, education professionals, and strategic health leaders.

### 2.2 Domains

**Fig. 1:**
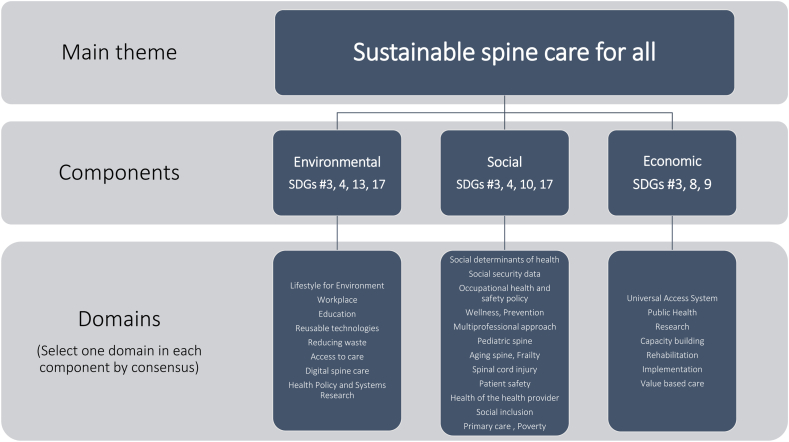
Components of domains.

The SPINE20 recommendations were built on specific domains selected by consensus. All domains were reviewed and updated by December 2024 and stratified into three groups by the SPINE20 program task forces (Environmental components, social components, economic components: Fig. 1) to achieve SPINE20's main theme.

### 2.3 Selection of domains

An online real-time Delphi consensus meeting was held on January 29, 2025, to select one domain from each of the three components. Two facilitators (KT from Japan and SA from Saudi Arabia) facilitated the meeting, and 39 international, multidisciplinary experts from 28 spine societies participated. A week prior to the Delphi consensus meeting, participants scored each domain (from 1 to 10) to provide an initial prioritization. In the Delphi consensus process, a maximum of four rounds of voting was allowed per group. Domains from any group that reach the 60 % level of agreement within four rounds were adopted (Table 2, Table 3). The four domains making the final selections were: “Sustainability”, “Public Health”, “Occupational health and safety policy”, and “Capacity building”. “Sustainability” was designated as a central concept in recognition of the SPINE20 2025 summit theme.

**Table 2:** Results of pre-scoring.

Components	Domains with average point (min:0, max:10)
Environmental	Education: 7.97, Public Health: 7.90, Access to care: 7.65 Workplace: 7.26, Avoiding futile treatment: 7.23, Reducing waste: 7.10, Health Policy and Systems Research: 7.03, Reusable technologies: 6.84, Digital spine care: 6.16
Social	Multiprofessional approach: 8.35, Occupational health and safety policy: 7.39, Prevention: 7.39, Aging spine: 7.23 Primary care: 7.19, Patient safety: 6.90, Social determinants of health: 6.77, Frailty: 6.52, Poverty: 6.52, Social inclusion: 6.19, Spinal cord injury: 6.03, Wellness: 6.00, Pediatric spine: 5.71, Social security data: 5.42
Economic	Rehabilitation: 8.03, Research: 7.74, Capacity building: 7.55, Implementation: 7.52, Value based care: 7.48, Health of the health provider: 7.23

**Table 3:** Results of Delphi consensus voting.

	1st vote	2nd vote	3rd vote	4th vote
**Environmental component**				
Education	31 %	48 %	40 %	28 %
Public Health	54 %	53 %	60 %	72 %
Access to care	15 %			

**Social component**				
Multiprofessional approach	46 %	52 %	40 %	32 %
Occupational health and safety policy	35 %	48 %	60 %	68 %
Prevention	19 %			

**Economic component**				
Rehabilitation	42 %	52 %	44 %	36 %
Research	23 %			
Capacity building	35 %	48 %	56 %	64 %

### 2.4 Development of recommendations

The development of the 2025 SPINE20 recommendations was led by domain-specific writing groups, each comprising three experts appointed by the SPINE20 Scientific Committee. The appointed writers were as follows:

Sustainability: IV (South Africa), TB (Germany), KK (India)

Public Health: RD (South Africa), CT (Brazil), CR (Italy)

Occupational Health and Safety Policy: LR (UK), SA (Saudi Arabia), RY (Chile)

Capacity Building: MC (United States), KK (Japan), FT (South Africa)

Domain leads engaged additional specialists as needed to draft and justify proposals. Preliminary outlines were reviewed and refined in fortnightly Scientific Task Force meetings. Through a collaborative, iterative process—integrating expert feedback across fields—the recommendations were shaped to be scientifically robust, contextually relevant, and policy-actionable.

### 2.5 Publication of recommendations

The proposed recommendations and their underpinning rationale were reviewed by partner societies before they were made available to the public. The recommendation statements and supporting rationales were published on the SPINE20 website (https://spine20.net) 7 days before the SPINE20 summit which took place on October 10–11, 2025, in Cape Town, South Africa. Public comments were collected via the website and considered, as the recommendations were refined. These recommendations were then discussed at the SPINE20 Summit 2025, allowing participants to debate the recommendations and suggest further modifications. The recommendations were voted on at the SPINE20 summit, and only those that were approved were officially published as SPINE20 2025 Recommendations.

The complete set of SPINE20 2025 Recommendations, along with their supporting rationales, is publicly available on the official SPINE20 website (https://spine20.net), ensuring open access for policymakers, clinicians, and researchers worldwide. During the SPINE20 Summit 2025 held in Cape Town, South Africa, the recommendations were officially presented to Mireille Mary Wenger, Provincial Minister of Health and Wellness, Western Cape Government. Following the summit, an official communication from the Western Cape Ministry of Health and Wellness confirmed formal acknowledgment of receipt of the document.

This paper serves as a summary of the above-mentioned recommendations.

## 3 Results

### 3.1 Domain: sustainability

SPINE20 recommends G20 countries to implement sustainable evidence-based spine care models drawing on successful global programs considering particularly registries, incentivized health targets and public-private partnerships.

#### 3.1.1 Related SDGs

Relevance to United Nations SDGs: 3) Good health and well-being; 8) Decent work and economic growth; 10) Reduced inequalities; 17) Partnerships for the Goals.

#### 3.1.2 Context to the domain

Spine disorders are among the leading global causes of disability, with low back pain alone affecting over 620 million people and resulting in immense economic costs from absenteeism and healthcare expenditures (GBDLBP, 2023; Wu et al., 2020). The SPINE20 2025 initiative emphasizes the need for evidence-based and sustainable spine care systems that adapt global best practices to national realities (AlEissa et al., 2021; Costanzo et al., 2022; Darwono et al., 2022; Chhabra et al., 2023; Menezes et al., 2025). The focused domains of Public Health, Occupational Health and Safety Policy, and Capacity Building are essential pillars for achieving equitable and sustainable care models.

#### 3.1.3 Problem

Spinal disorders are a leading cause of disability-adjusted life years (DALYs) in the world and also South Africa, leading to high levels of work absenteeism and welfare dependence (GBDLBP, 2023; Wu et al., 2020). Mining, construction, and transport sectors face particularly high injury rates due to insufficient ergonomic measures (Okello et al., 2020). Rehabilitation services are underdeveloped and often unaffordable, and care pathways remain fragmented between public and private providers (Fay and Black, 2024; Mazibuko et al., 2024). Key challenges to advancing spine health include the absence of national spine registries, which limits evidence-based policymaking and quality monitoring; economic barriers, where high out-of-pocket costs for rehabilitation and imaging deter timely access to care; and persistent workplace risks due to insufficient enforcement of occupational health standards, leading to elevated injury rates (van Hooff et al., 2015; Pascucci et al., 2023; Ranjbar Hameghavandi et al., 2024). Furthermore, shortages in physiotherapists, occupational therapists, and other non-surgical care providers constrain service capacity. Low public awareness—characterized by misconceptions favoring prolonged rest and over-medication—further delays recovery and increases the risk of chronicity (de Witt et al., 2024).

#### 3.1.4 Potential solutions

SPINE20 recommends an integrated approach combining prevention, early diagnosis, effective treatment, and rehabilitation.

• National/Regional Spine Registries:

Developing a national spine registry would enable outcome tracking, risk assessment, and data-driven policy decisions (van Hooff et al., 2015; Pascucci et al., 2023; Ranjbar Hameghavandi et al., 2024). Models like Sweden's Swespine, Eurospine's Spine Tango and the Japanese national registry (JOANR) have demonstrated improved quality and cost control (Stromqvist et al., 2013; Taneichi et al., 2023). Such registries contribute to the sustainability of spine care by promoting efficiency, transparency, and continuous improvement.

• Incentivized Health Targets:

Governments can introduce outcome-based incentives and bundled rehabilitation payments for healthcare facilities, rewarding adherence to evidence-based guidelines and improvements in clinical back pain outcomes (de Bruin et al., 2011; Einav et al., 2019). The incentives foster sustainable spine care by aligning quality improvement with long-term resource efficiency. Such platforms could also host open-access education, tele-rehabilitation services, and AI-powered screening tools, expanding reach and equity in care delivery (Abernethy et al., 2022).

• Public Health and Occupational Interventions:

Details are provided in a separate section.

• Capacity Building:

Details are provided in a separate section.

• Public-Private Partnerships:

Collaboration with tech companies, insurers, and employers can accelerate registry development, digital infrastructure, and research on preventive strategies (Mazibuko et al., 2004; Kula and Fryatt, 2014). Such partnerships enhance the sustainability of spine care by pooling resources, fostering innovation, and ensuring long-term system resilience.

#### 3.1.5 Expected outcomes

Implementing these measures would.

• Improve Access and Equity: Tele-rehabilitation and reward-based platforms would bridge urban–rural divides.
• Enhance Data-Driven Policy: Registries would allow benchmarking and quality improvement.
• Boost Adherence and Engagement: Incentive-based digital platforms motivate patients to follow rehabilitation plans.
• Reduce Disability and Costs: Workplace reforms and early interventions would lower absenteeism and long-term care expenses.
• Strengthen Workforce: Expanded training and interdisciplinary education would address skill gaps.
• Promote Innovation: Public-private partnership and digital platforms would support scalable, cost-effective solutions.

By aligning these strategies with the UN Sustainable Development Goals (SDG 3, 8, 10 and 17), South Africa and other G20 countries can lead the way in developing sustainable spine care systems that reduce disability, enhance economic productivity, and ensure equitable access to care.

### 3.2 Domain: public health

SPINE20 recommends G20 countries to integrate spine health into public health and primary care health policies by addressing the prevention and management of both communicable and non-communicable diseases, and strengthening public–private partnerships to achieve sustainable spine care.

#### 3.2.1 Related SDGs

Relevance to United Nations SDGs: 3) Good health and well-being; 8) Decent work and economic growth; 10) Reduced inequalities.

#### 3.2.2 Context to the domain

Over the past decades, global efforts to combat infectious diseases such as HIV and tuberculosis (TB) have contributed to building strong and resilient public health systems in many regions. Within this broader public health architecture, primary care functions as the front line: ward-based outreach teams, community health workers, and Ideal Clinic standards enable reliable delivery of services, supplies, and health messages from national to community level (Policy Framework and Strategy for; What is an Ideal Clinic, 2025). By providing first-contact, accessible, and continuous care, primary care clinics not only treat individuals but also contribute directly to population-level goals: prevention, surveillance, and early response to health threats. This function—clinical care for individuals and population health impact through scale and integration—explains why strengthening primary care is indispensable for public health progress. For instance, ongoing health system reforms worldwide aim to advance universal health coverage by strengthening primary care and improving the strategic purchasing of essential services—establishing policy conditions that support the inclusion of rehabilitation, prevention, and spine health within routine services and care quality standards (National Health Insurance, 2025; National Health Insurance, 2023; WHO South Africa Welcomes Signing, 2024).

#### 3.2.3 Problem

Musculoskeletal and spine conditions remain under-recognized in health benefits, metrics, and clinic workflows (GBDLBP, 2023; GBDOMD, 2023). Patients commonly cycle through episodic visits and analgesics without active self-management, early rehabilitation, or clear referral triggers. The burden is amplified by multifaceted risk environments seen globally, with particularly significant effects in low- and middle-income countries: heavy manual work (mining, logistics, agriculture, construction), long-distance commuting, road traffic injuries, and constrained access to conservative care (Morris et al., 2018). Older adults and those in the casual labor sector face additional barriers to rehabilitation and return-to-work. Without integration into primary care, spine conditions silently erode quality of life, workforce participation, and fiscal sustainability. (WHO: Rehabilitation)

#### 3.2.4 Potential solutions

1) Integrate first-line spine care into primary and public health workflows through brief screening, red-flag recognition, culturally sensitive self-management tools, and digital resources for nurses and community health workers.
2) Establish a tiered care pathway with clear referral thresholds: community and clinic levels focus on education, reassurance, activity restoration, and simple analgesia; district hospitals provide multidisciplinary assessment, limited imaging, and rehabilitation; referral centres manage complex surgical cases with integrated peri-operative rehabilitation.
3) Embed rehabilitation in the primary-care benefits package, aligned with WHO Rehabilitation 2030, ensuring continuity from prevention to recovery. (WHO)
4) Strengthen workforce capacity through rapid upskilling, mentorship hubs led by rehabilitation specialists, tele-supervision for rural areas, and standardised documentation to monitor function and return-to-work progress.
5) Foster public–private partnerships to expand access, align incentives, and leverage innovation for scalable service delivery and data systems.
6) Measure what matters by adding a minimal musculoskeletal indicator set to routine dashboards—tracking first-line care adherence, functional recovery, and timely referral—disaggregated by district, occupation, and sex/age to guide resource allocation.

#### 3.2.5 Expected outcomes

Over the medium term, the initiative aims to reduce disability and absenteeism, improve pain and function outcomes, and enhance workforce participation while increasing system efficiency through bundled benefits and targeted rehabilitation. Safer workplaces and community environments will emerge through embedded ergonomic and injury-prevention policies.

In the longer term, an integrated, equitable spine-care pathway will sustain productivity, lower costs, and provide a scalable model for other G20 countries to embed spine health within primary care and public health systems—transforming silent disability into measurable functional recovery and supporting universal health coverage.

### 3.3 Domain: Occupational Health & Safety Policy

SPINE20 recommends that G20 countries implement evidence-informed, work-focused interventions that address employee and workforce factors early, to reduce the social and economic impact of work loss and increase employability for people with spine disorders.

Relevance to United Nations SDGs: 3) Good health and well-being; 8) Decent work and economic growth; 10) Reduced inequalities; 16) Peace, justice, and strong institutions.

#### 3.3.1 Context

Work loss through sickness absence and presenteeism is increasing, with back pain being the leading cause (Gabbay et al., 2011; Chetty, 2017). Little prevention research exists, and there is no universal approach to managing spine disorders (Foster et al., 2018). Furthermore, although evidence-based guidelines exist, they are based on trials almost exclusively from high-income countries, largely developed without service-user involvement and are inconsistently applied in practice by health care providers (Chetty, 2017; Foster et al., 2018). The lack of implementation delays recovery and return to work, with unnecessary imaging, polypharmacy, bed rest, specialist referrals and passive treatments and underuse of biopsychosocial approaches (Chetty, 2017; Foster et al., 2018; Owens et al., 2019; Ryynanen et al., 2021).

#### 3.3.2 Problem

Multiple factors contribute to work loss from spine disorders including the individual employee, their job role and the culture and practices within the workplace. Specific to the employee, negative beliefs about their recovery and fears associated with pain or reinjury are strong predictors of absence and prolonged symptoms (Rashid et al., 2017; Becker and Childress, 2019). Rising rates of sedentary practices at work and leisure, increased workloads and stress, all impact recovery from spine disorders (Lurati, 2016). Meanwhile, physically demanding jobs that include repetitive lifting, bending twisting and static postures have been identified as physical risk factors and 3-D jobs ‘dirty, dangerous and demanding’ are challenging in general, due to unequal representation of racial and ethnic minority workers, often working with employment uncertainty, low wages and poor or dangerous working conditions (Chetty, 2017; Becker and Childress, 2019; Lurati, 2016; Rosenberg et al., 2021; Quandt et al., 2013). The culture and practices within the workplace further influence outcomes. Where job insecurities exist, fear of job loss can result in presenteeism as employees attend work despite their symptoms, which can worsen their condition and, in many societies, the workforce is aging and thus, more employees are living with long-term conditions and degenerative pathologies (Luites et al., 2022).

#### 3.3.3 Potential solutions

To optimize a positive outcome, early identification of symptoms is required, with fast-track access to appropriate support and care (Gabbay et al., 2011; Foster et al., 2018; Becker and Childress, 2019). Culturally adapted, plain language materials should be provided to support shared decision-making and provide clarity about work options and likely prognosis (Amundsen et al., 2024). An important component is person-centered reassurance, education about the non-dangerous nature of most spine disorders and self-management advice that includes advice to keep active, build strength, fitness and confidence and identify pacing strategies, is key (Foster et al., 2018; Becker and Childress, 2019; Slaughter et al., 2015).

Guidelines recommend a stepped-care model to guide return to work, escalating care to include workplace modifications, physical therapy, or cognitive behavioral therapy if there is no improvement by 6–12 weeks, considering multidisciplinary rehabilitation if barriers persist (Lurati, 2016; Luites et al., 2022). It is essential to foster co-ordination between health care providers, employers and insurers to best support the employee with shared action plans across healthcare and workplace settings, addressing fear-avoidance beliefs (Gabbay et al., 2011; Becker and Childress, 2019; Hellman et al., 2015). Implementation of guidelines can be measured through audit, to identify gaps in healthcare provision or in the workplace, and training needs (Owens et al., 2019). Workplace modifications may include ergonomic adaptations to equipment, as well as role adjustments to reduce physical demands, workplace exercise programs, and flexibility in hours/breaks (Becker and Childress, 2019; Lurati, 2016; Luites et al., 2022; Reneman et al., 2024). It is vital to develop open communication between employees and employers about any reasonable adjustments, with a culture of empathy and support, including follow-up reviews to discuss progress.

#### 3.3.4 Expected outcomes

SPINE20 recommends a PREMIUM approach to Occupational Health and Safety Policy.

• P = Prevent sickness absence in the workplace
• R = Recognize symptoms of musculoskeletal disorders early
• E = Educate including advice on self-management and keeping active
• M = Modify work practices
• I = Implement guidelines for managing musculoskeletal disorders
• U = Unite healthcare systems, employers and employees by effective communication
• M = Monitor adherence

### 3.4 Domain: capacity building

SPINE20 recommends that G20 countries prioritize building capacity in spinal cord injury care by adopting evidence-based interventions such as the global initiatives supported by WHO in low- and middle-income countries and aligned with the WHO Rehabilitation 2030 Call to Action.

#### 3.4.1 Relevance to United Nations SDGs

3) Good health and well-being; 10) Reduced inequalities; 16) Peace, justice and strong institutions; 17) Partnerships for the goals.

#### 3.4.2 Context to the domain

Spinal Cord Injury (SCI) presents a major global health challenge, especially in low- and middle-income countries where health systems are not adequately equipped to respond. For instance, in sub-Saharan Africa, including South Africa, the epidemiology of SCI significantly differs from that in developed countries with the trauma-dominated profiles. While traumatic causes like motor vehicle accidents accounted for 44.6 % of SCIs, non-traumatic cases are increasingly recognized with infectious spondylitis, particularly due to HIV and tuberculosis, being a leading cause (Sothmann et al., 2015). The incidence of infection-related SCIs has risen, correlating with the HIV epidemic and the prevalence of tuberculosis (Marais et al., 2018). Both traumatic and non-traumatic SCI contribute to the burden (Covell et al., 2023) and the high incidence rate of 75.6 per million in South Africa underscores the need for effective community reintegration strategies (Nizeyimana et al., 2025).

#### 3.4.3 Problem

The emergence of spinal tuberculosis as a significant cause of SCI and myelopathy highlights the need for ongoing surveillance and targeted interventions in this demographic (Marais et al., 2018). Conversely, while infectious causes are prominent in low- and middle-income countries, the global trend still shows trauma as the primary cause of SCIs in developed nations, emphasizing the need for tailored prevention strategies in different contexts. Delayed diagnosis, inappropriate surgical indications and limited access to specialized rehabilitation services exacerbate the problem. Furthermore, social determinants of health, such as income inequality and rural location, significantly impact SCI outcomes in low- and middle-income countries (Covell et al., 2023). Despite policy emphasis on comprehensive rehabilitation, community reintegration remains inadequate (Nizeyimana et al., 2025).

#### 3.4.4 Potential solutions

A strengthened capacity-building initiative for SCI care, aligned with WHO's Rehabilitation 2030 goals, will yield transformative outcomes for health systems and countries contexts. Integrating the International Spinal Cord Society (ISCoS) Standards Toolkit into national rehabilitation frameworks will enable the operationalization of high-quality, context-sensitive care practices and help bridge critical resource and knowledge gaps in SCI rehabilitation (Middleton et al., 2014).

By emphasizing the training and deployment of physical medicine and rehabilitation specialists, national health systems will build multidisciplinary teams equipped to address the complex biopsychosocial needs of people with SCI. Research underscores the role of physical medicine and rehabilitation in improving long-term functional outcomes, supporting community reintegration, and optimizing health system efficiency (Middleton et al., 2014; Stucki et al., 2017). Investment in physical medicine and rehabilitation training is also key to ensuring sustained implementation of SCI-specific clinical standards and aligning national curricula with global benchmarks.

Evidence from recent work demonstrates how integration of clinical research, practice, and implementation tools have advanced SCI care in diverse settings through improved monitoring, patient-centered indicators, and quality assurance systems (Middleton et al., 2014). These measures enhance outcome tracking and resource allocation, and promote health equity. This harmonization with the Rehabilitation 2030 vision ensures that capacity building is not only clinically sound, but systemically scalable and sustainable.

#### 3.4.5 Expected outcomes

The expected impact includes improved early diagnosis and timely referral, increased access to specialized rehabilitation services, and enhanced quality of life for people living with SCI. Furthermore, investment in rehabilitation professionals will reduce secondary complications, hospital readmissions, and economic losses due to long-term disability. These outcomes support the United Nations Sustainable Development Goals by fostering inclusion, reducing inequality, and strengthening health systems. When effectively implemented, these efforts can significantly improve diagnosis rates, care access, rehabilitation outcomes, and overall health system resilience. Improving rehabilitation services is crucial for enhancing individual well-being and economic contribution (Morris et al., 2021).

## 4 Conclusion

Spine disorders are a major cause of disability worldwide, demanding coordinated strategies to ensure equitable and sustainable care. SPINE20's 2025 recommendations—focused on Sustainability, Public Health, Occupational Health and Safety Policy, and Capacity Building—propose evidence-based actions such as national spine registries, integrated primary care, workplace interventions, and workforce development. Officially presented to the Western Cape Government and formally acknowledged by its Minister of Health and Wellness, these recommendations reinforce policy relevance and practical applicability. From 2025 onward, SPINE20 has also begun issuing a complementary “Call for Action” series that deepens and disseminates key messages from previous recommendations to accelerate their implementation. By embedding spine health within public health agendas and investing in sustainable rehabilitation systems, G20 countries can reduce disability, strengthen workforce participation, and promote inclusive growth. Aligned with the United Nations Sustainable Development Goals (SDG 3, 8, 10, 16, and 17), these collective efforts place spinal health at the core of global well-being, equity, and sustainability.

## Consent to participate

All authors have read and approved the final version of the paper.

## Availability of data and material

The datasets generated during the current study are available from the corresponding author on reasonable request.

## Code availability

Not applicable.

## Ethics committee approval

Not applicable to the current policy paper.

## Author contributions

AV, and KT managed in developing the paper all steps. SA, TB, MC, RD, KKa, KKi, LR, CR, FT, CT, and RT led the drafting of this paper in collaboration with the other authors and were part of the team that coordinated the production of papers. BB, MC, QL, DL, and AO closely revised many sections. Thereafter all authors contributed to all sections of the paper and edited it for key intellectual content. All other authors have read and provided substantive intellectual comments to the draft and have approved the final version of the paper.

## Consent for publication

All authors give our consent for the publication of identifiable details to be published in the Global Spine Journal.

## Funding

No funds were obtained.

## Conflicts of interest statements

Nothing to disclose.
